# Effectiveness, Usability, and Satisfaction of a Self-Administered Digital Intervention for Reducing Depression, Anxiety, and Stress in a University Community in the Andean Region of Peru: Randomized Controlled Trial

**DOI:** 10.2196/71465

**Published:** 2025-10-15

**Authors:** Rosario Yslado-Méndez, Stefan Escobar-Agreda, David Villarreal-Zegarra, Wilfredo Manuel Trejo Flores, Junior Duberli Sánchez-Broncano, Ana Lucia Vilela-Estrada, Jovanna Hasel Olivares Córdova, C Mahony Reategui-Rivera, Claudia Alvarez-Yslado, Leonardo Rojas-Mezarina

**Affiliations:** 1 Facultad de Ciencias Médicas, Universidad Nacional Santiago Antúnez de Mayolo, Av. Centenario 200, Huaraz, 02002, Peru, +51 042640020; 2 Unidad de Telesalud, Facultad de Medicina, Universidad Nacional Mayor de San Marcos, Lima, Peru; 3 Digital Health Research Center, Instituto Peruano de Orientación Psicológica, Lima, Peru; 4 Universidad Científica del Sur, Lima, Peru; 5 Facultad de Ciencias, Universidad Nacional Santiago Antúnez de Mayolo, Huaraz, Peru; 6 Escuela de Posgrado, Universidad San Ignacio de Loyola, Lima, Peru; 7 Facultad de Ciencias Sociales, Educación, Comunicación, Universidad Nacional Santiago Antúnez de Mayolo, Huaraz, Peru

**Keywords:** digital service, university community, mental health, depression, anxiety, stress

## Abstract

**Background:**

University communities, especially in low-resource settings like Peru’s Andean region, experience high rates of depression, anxiety, and stress, which harm academic performance and well-being. Traditional mental health services often remain inaccessible, necessitating scalable, self-guided solutions. While digital mental health interventions have shown promise broadly, evidence is scarce for fully self-administered platforms in low-resource university environments.

**Objective:**

We evaluated the efficacy of a self-administered digital mental health service in members of a public university to reduce symptoms of depression, anxiety, and perceived stress.

**Methods:**

We conducted a double-blind, parallel-group randomized controlled trial with 1:1 allocation to the digital mental health self-care service or a 30-day waiting-list control. We recruited 427 participants (students, teachers, and administrative staff) in May-June 2024, reporting mild to moderate symptoms of depression, anxiety, and stress. Participants were randomized via simple randomization and blinded through automated platform assignment. The intervention comprised 6 sequential 5-day modules grounded in acceptance and commitment therapy, mindfulness, and behavioral activation, delivered via videos, daily text prompts, workbooks, and a responsive chatbot. SMS and WhatsApp (Meta) reminders promoted adherence. Depressive symptoms (Patient Health Questionnaire-9), anxiety symptoms (Generalized Anxiety Disorder-7), and perceived stress (PSS-10) were assessed at baseline and immediately post intervention (day 30). Secondary outcomes in the intervention arm included usability (Computer System Usability Questionnaire), satisfaction (Client Satisfaction Questionnaire-8), and subjective commitment (Twente Engagement with Ehealth Technologies Scales). Analysis of covariance (ANCOVA) adjusted for baseline scores, and multivariate ANCOVA accounted for correlations among outcomes. Effect sizes were quantified using Cohen *d* and partial epsilon-squared (ε²_p_).

**Results:**

Of 427 randomized, 85 (19.9%) completed all assessments (intervention: n=30; control: n=55). Baseline demographic and clinical characteristics were comparable between groups. Post intervention, the digital mental health self-care service group exhibited significantly greater reductions in mean Patient Health Questionnaire-9 scores (mean difference 2.78; Cohen *d*=0.64; *P*=.006), Generalized Anxiety Disorder-7 scores (mean difference 2.13; Cohen *d*=0.56; *P*=.015), and PSS-10 scores (mean difference 4.08; Cohen *d*=0.69; *P*=.003) than controls. ANCOVA confirmed robust group effects for depression (*F*₁,₈₂=9.78; *P*=.002; ε²_p_=0.31) and anxiety (*F*₁,₈₂=8.28; *P*=.005; ε²_p_=0.32), with a trend toward stress reduction (*F*₁,₈₂=3.73; *P*=.057; ε²_p_=0.46). Multivariate ANCOVA demonstrated a significant multivariate effect (F₄,₁₂=7.23; *P*=.015). Among intervention completers, 100% scored below the Client Satisfaction Questionnaire-8 satisfaction threshold (<24), 60% rated platform usability as low (Computer System Usability Questionnaire<64); yet, 96.7% reported high subjective commitment (Twente Engagement with Ehealth Technologies Scales ≥18), indicating strong engagement despite interface challenges.

**Conclusions:**

A self-administered digital self-care service effectively reduced depression, anxiety, and stress symptoms in a Peruvian university community. High user commitment underscores the platform’s relevance, while low satisfaction and usability necessitate interface optimization—streamlined navigation, adaptive personalization, and feedback mechanisms—to enhance user experience and support scalable implementation in low-resource educational settings.

## Introduction

Mental health problems generate a high burden worldwide, contributing losses of productivity and affecting the global economy [1]. Thus, their presence in university students triggers poor academic performance and dropout, which negatively affects their chances of getting a gainful job [2,3]. This also affects teaching staff, whose well-being is linked to the mental health of students because of their close relationship [4]. One of the countries that most clearly evidences this problem is Peru, where 85% of the university community—comprising teachers, students, and administrators—report problems such as anxiety, stress, and violence [5]. This situation is caused, among other factors, by preexisting mental health problems in incoming students, which are aggravated in the Peruvian university environment, even resulting in suicide attempts [6]. Specifically, in the Andean region, where inhabitants have a greater predisposition to depression [7], college students are vulnerable to developing depression, anxiety, and stress [8].

In response to this situation, digital mental health interventions (DMHI), defined as the use of technology for the prevention and treatment of mental health conditions [9], emerge as a promising alternative. DMHI have been shown to be effective in reducing these conditions among university students, while also improving access to mental health services [9,10]; nevertheless, a recent systematic review of 89 college-student trials highlighted persistent gaps in usability reporting and real-world implementation [11]. Beyond their potential impact in Peru, the rollout of DMHI in educational environments is supported by the interest of health authorities, who emphasize the need for youth-centered services [12], and the increasing use of digital services accelerated by the COVID-19 pandemic [13]. In fact, some universities here have implemented interventions and containment measures through strategic actions that combine in-person and virtual primary mental health care services for the broader university community [14].

However, the success of DMHI can be hindered by several factors, including lack of contextualization [15], nonintuitive interface design that leads to low usability, satisfaction, and applicability [16], as well as poor adoption and acceptability [17]. Evidence suggests that the development of these interventions should be carried out with the end user, particularly when targeting young populations [18]. In Peru, various favorable experiences have been reported in the implementation of DMHI delivered via text messaging [19], telephone assistance [20], and remote support by a professional [21]; however, evidence is limited regarding the use of self-management platforms in higher education settings. Accordingly, the present study aimed to evaluate the efficacy of a digital self-care mental health service to reduce the symptoms of depression, anxiety, and perceived stress in the community of a public university in the Andean region of Peru. In addition, we aimed to understand its usability, subjective commitment, and user satisfaction.

## Methods

### Study Design

**Figure 1.**
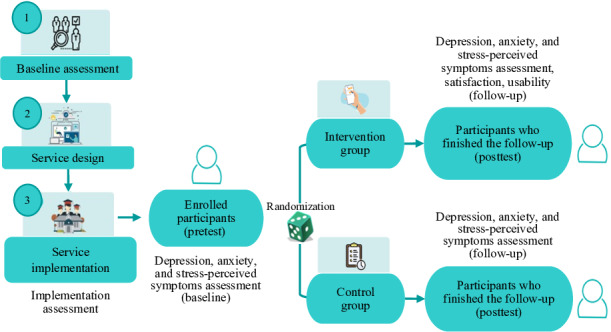
Parallel randomized controlled trial design.

We conducted a double-blind, parallel-group randomized controlled trial (RCT) with a superiority hypothesis and equal allocation (1:1) to a digital mental health self-care service (DMHSS) or a waiting-list control. Assessments were performed at baseline and 1 month after the intervention. The trial protocol was preregistered on the Open Science Framework (6AQSB), and the reporting follows the CONSORT-EHEALTH (Consolidated Standards of Reporting Trials of Electronic and Mobile Health Applications and Online Telehealth) extension [22]. An overview of the trial procedures is shown in Figure 1.

### Participants

Members of the university community were invited from May to June 2024 to complete a baseline mental health screening via the self-care platform: professors (n=444), administrative staff (n=266), and students (n=7710) at a public Peruvian Andean university. Those aged ≥18 years who responded to the pretest and exhibited mild to moderate symptoms of depression, anxiety, or perceived stress were eligible for inclusion. Exclusion criteria were refusal of virtual informed consent; lack of prior experience with any virtual health service; being on leave or having reserved enrollment during the study period; current psychotherapy or psychopharmacological treatment for depression, anxiety, or stress; a prior diagnosis of severe mental health pathology; or scoring in the severe range on the baseline assessment.

### Preintervention Phases

#### Formative Assessments

We conducted formative qualitative and quantitative evaluations to understand user preferences, platform requirements, and the prevalence of mental health problems and related factors in this population. In addition, the quantitative data allowed us to assess the psychometric properties of the mental health instruments (factorial validity and internal consistency were adequate).

#### Design and Development of the Digital Mental Health Self-Care Service

The design and deployment of the DMHSS involved determining the workflows and information to be delivered and building a web platform tailored to user needs identified in the formative evaluation. The platform integrated audiovisual and textual materials related to the intervention, a pre- and posttest module with automatic grading for assessment purposes, and a chatbot that responded to frequently asked questions about the platform’s use. User manuals and tutorial videos were also included to facilitate adoption. The platform was developed using Vue.js for the user interface, Dialogflow for the chatbot, MySQL for database management, and the Laravel framework for backend services, following the Scrum Agile software development methodology.

Prior to its implementation, the platform was tested by the technical team and trained personnel to verify compliance with quality, security, data protection, and data flow reliability standards. In addition, an implementation checklist was used to assess the platform’s operational performance through a pilot test with 30 participants from the study population. This sample size was selected to ensure early feedback on functionality and usability while maintaining operational feasibility during the preimplementation phase.

### Intervention

**Figure 2.**
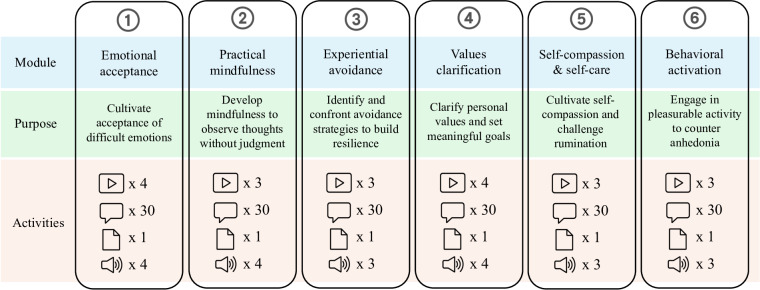
Flowchart of the 6-module digital mental health self-care service.

We refer to our self-administered digital intervention throughout as the DMHSS, branded as “Amyga” for the student interface [23]. The DMHSS was developed and overseen by a clinical psychologist specializing in cognitive-behavioral psychotherapy (CBT) to ensure fidelity to evidence-based principles. It comprises 6 self-administered, 5-day modules grounded in a contextual-behavioral model (Figure 2). Each module combined 3‐4 brief (5‐8 min) videos (with matching audio tracks), 20‐30 daily text prompts, and a 3‐ to 8-page PDF workbook, all adaptively tailored to users’ baseline Patient Health Questionnaire-9 (PHQ-9) and Generalized Anxiety Disorder-7 (GAD-7) scores. The modules were organized into 3 pairs by psychotherapeutic technique: modules 1 and 4 applied acceptance and commitment therapy (ACT) to foster emotional acceptance and clarify personal values [24]; modules 2 and 5 used mindfulness practices to cultivate nonjudgmental present-moment awareness and stress tolerance [25]; and modules 3 and 6 targeted experiential avoidance through gradual exposure and behavioral activation to build resilience and engagement in meaningful activities [26]. Detailed content and resource quantities for each module are provided in Table S1 in Multimedia Appendix 1.

In each module, the video icon denotes short tutorials that blend theoretical background with practical examples; the daily-message icon marks brief prompts sent each day to reinforce learning and encourage reflection; the document icon indicates downloadable documents with step-by-step instructions and written exercises; and the audio icon represents narrated versions of the tutorials and exercises for offline or hands-free use.

We reinforced engagement by sending SMS or WhatsApp reminders to participants at the beginning, middle, and end of each module. In addition, reminders were sent to participants who did not complete the activities in the estimated time, in order to encourage compliance with the established deadlines. Control-group participants were placed on a 30-day waiting list; after this period, they received full access to the DMHSS. All participants also received information on standard university mental health services.

### Variables and Measurement Instruments

#### Main Outcomes

The study had 3 central outcomes: depressive symptoms, anxious symptoms, and perceived stress. Depressive symptoms were measured using the PHQ-9, a 9-item instrument validated in the Peruvian university population [27], scored from 0 (not at all) to 3 (nearly every day). Anxiety symptoms were measured using the GAD-7, a 7-item instrument validated in the Peruvian university population [28] with items scored 0‐3 on a Likert scale. Perceived stress was measured with the Perceived Stress Scale-10 (PSS-10; 10 items, scored 0‐3), validated in the Peruvian population [29]. All 3 scales were administered at baseline (day 0) and immediately postintervention (day 30).

#### Secondary Outcomes

Participants in the intervention group who completed all modules were assessed for satisfaction, usability, and subjective engagement with the digital service. Usability was measured with the Computer System Usability Questionnaire (CSUQ), a 16-item questionnaire scored on a 7-point Likert scale. Subjective commitment was measured with the Twente Engagement with Ehealth Technologies Scale (TWEETS) questionnaire (9 items on a 5-point Likert scale). Finally, satisfaction was assessed using the Client Satisfaction Questionnaire-8 (CSQ-8; 8 items). Scores below each instrument’s cutoff point defined a “low” category: CSQ-8<24 for satisfaction, CSUQ<64 for usability, and TWEETS <18 for subjective commitment.

#### Covariates

Covariates included sex, age, device preferences (phone, PC, and tablet), mobile internet access, participant role (student, teacher, and administrator), and history of mental health diagnosis.

#### Sample Size

We based our sample size calculation on the minimum number of participants required to conduct our primary analysis, which aims to detect differences between the control and intervention groups in their pre- and postintervention scores. Accordingly, we applied a one-way analysis of covariance (ANCOVA) model with 2 arms (control and experimental) and 2 time points (pre and post). We set statistical power at 80%, *α*=.05, and assumed a pre–post correlation of .7 and a medium effect size (η²=0.20), following Shieh’s approach [30]. This procedure yielded a required minimum sample of 102 participants, corresponding to 51 participants per arm. We adopted the medium effect size estimate on the basis of a previous randomized clinical trial that used ANCOVA with PHQ-9 and GAD-7 outcomes in a DMHI [31].

#### Randomization and Blinding

Randomization sequences were generated using the randomizeR package, applying simple randomization to allocate participants 1:1 to control or intervention groups, with 10,000 allocations produced.

Investigators and analysts were blinded. The platform automatically assigned participants; group allocation remained concealed until final analysis and manuscript drafting to prevent bias.

### Procedures

In collaboration with the university welfare department, we ran an advertising campaign to recruit students, teachers, and administrative staff via campus announcements and online registration. Eligible individuals were then randomized to the intervention or waiting-list control. The intervention group engaged with the 6-module DMHSS over the next 30 days, while control participants remained on the waiting list and received access afterward. Both groups completed the postintervention assessment immediately on day 30 using the same online instruments.

### Statistical Analysis

Continuous variables are presented as mean (SD) or median (IQR); categorical variables as frequencies and percentages. Normality was assessed with Shapiro–Wilk, and independent-samples *t* tests were applied when appropriate. To isolate the effect of the intervention on posttest scores while accounting for baseline differences, we performed ANCOVA, which adjusts each follow-up outcome for its corresponding pretest value. Intervention effects were quantified with Cohen *d* (0.20=small; 0.50=moderate; 0.80=large) and partial epsilon-squared (ε²_p_) (0.02=small; 0.15=moderate; 0.30=large) for intuitive purposes [32]. All analyses were performed in RStudio (Posit PBC) and Stata 17 (StataCorp LLC).

### Ethical Considerations

The study protocol was approved by the Universidad Nacional Santiago Antúnez de Mayolo Research Ethics Committee (report numbers 011‐2022 and 07‐2023). All participants provided electronic informed consent before taking part and were free to withdraw at any time without penalty. No financial or other compensation was offered for participation. To protect privacy and confidentiality, we assigned alphanumeric codes to each record, stored data on secure sockets layer–encrypted servers, and removed all personal identifiers after analysis in compliance with Peru’s data protection law (Number 29733). The study was conducted in accordance with the World Medical Association’s ethical principles [33].

## Results

**Figure 3.**
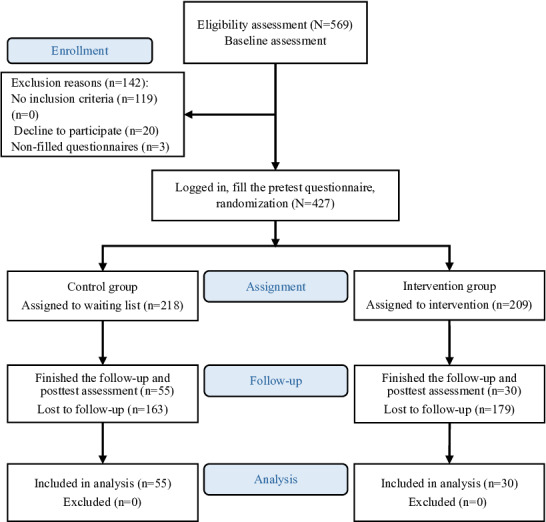
Study flowchart.

Of the 569 participants who registered on the self-care web platform, 119 were excluded for presenting severe symptoms, 20 for invalid data or ineligibility, and 3 for incomplete baseline questionnaires, resulting in 427 participants evaluated at baseline. Among these, 218 were randomized to the control group, and 209 to the intervention group. Ultimately, 85 (19.9%) completed the full intervention and postintervention assessments (Figure 3).

**Figure 4.**
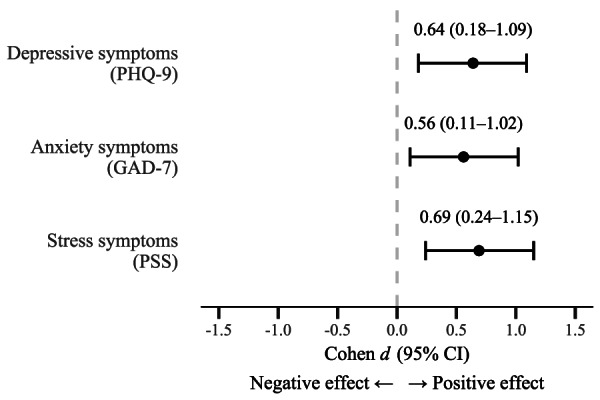
Between-group effect sizes (Cohen *d*) for depressive, anxiety, and stress symptoms. GAD-7: Generalized Anxiety Disorder 7; PHQ-9: Patient Health Questionnaire-9; PSS: Perceived Stress Scale.

Baseline characteristics—including age, sex, and initial mental health scores—were comparable between groups (Table 1). In Tables2  3, the scores of depressive symptoms, anxiety, and stress were compared before the intervention, and we observed that participants of the intervention group had lower stress scores (*P*=.02). Control group participants did not have a notable change in their scores of depressive symptoms, anxiety, or stress, but the participants of the intervention group did show a greater reduction in all scores. In addition, the latter showed effect measures considered large [32]. On the other hand, when comparing the control and intervention groups after the intervention, the results favored the intervention group with larger effect measures (all Cohen *d*>0.5). These standardized effect sizes and their 95% CI are summarized graphically in Figure 4.

**Table 1.** Characteristics of participants before the intervention.

Groups	Control		Intervention		*P* value
Age (years), mean (SD)	23.6 (7.3)		23.4 (6.7)		.78
Sex, n (%)					.80
Female	124 (56.6)		122 (57.8)		
Male	95 (43.4)		89 (42.2)		
Profession, n (%)					.52
Administrative staff	12 (5.5)		8 (3.8)		
Teacher	7 (3.2)		10 (4.7)		
Student	200 (91.3)		193 (91.5)		
Depressive symptoms, n (%)					.93
No symptoms	31 (14.2)		34 (16.3)		
Mild	95 (43.6)		87 (41.6)		
Moderate	56 (25.7)		55 (26.3)		
Severe	36 (16.5)		33 (15.8)		
Anxiety symptoms, n (%)					.60
No symptoms	49 (22.5)		48 (23)		
Mild	116 (53.2)		99 (47.4)		
Moderate	45 (20.6)		52 (24.9)		
Severe	8 (3.7)		10 (4.8)		
Stress symptoms, n (%)					.90
Mild	18 (8.3)		19 (9.1)		
Moderate	179 (82.1)		172 (82.3)		
Severe	21 (9.6)		18 (8.6)		

**Table 2.** Comparison of study groups according to assignment to the experiment and according to the time of intervention.

Study groups	Control (n=55)	Intervention (n=30)	Difference	Cohen *d* (95% CI)	*P* value
Before intervention, mean (SD)					
PHQ-9^a^ score	8.33 (4.91)	8.10 (4.83)	0.23	0.05 (−0.4 to 0.49)	.84
GAD-7^b^ score	6.53 (3.54)	6.47 (3.57)	0.06	0.02 (−0.43 to 0.46)	.94
PSS^c^ score	20.62 (5.10)	18 (4.42)	2.62	0.54 (0.08‐0.99)	.02
After intervention, mean (SD)					
PHQ-9 score	8.31 (4.73)	5.53 (3.56)	2.78	0.64 (0.18‐1.09)	.006
GAD-7 score	7.13 (4.03)	5 (3.24)	2.13	0.56 (0.11‐1.02)	.02
PSS score	19.55 (5.63)	15.47 (6.29)	4.08	0.69 (0.24‐1.15)	.003

a PHQ-9: Patient Health Questionnaire-9.

b GAD-7: Generalized Anxiety Disorder Assessment-7.

c PSS: Perceived Stress Scale.

**Table 3.** Comparison of study groups according to the time of intervention.

Study groups	Before intervention (n=219)	After intervention (n=85)	Difference	Cohen *d* (95% CI)	*P* value
Control group, mean (SD)					
PHQ-9^a^ score	8.33 (4.91)	8.31 (4.73)	0.02	0.003 (−0.37 to 0.38)	0.98
GAD-7^b^ score	6.53 (3.54)	7.13 (4.03)	−0.60	−0.16 (−0.53 to 0.22)	0.19
PSS^c^ score	20.62 (5.10)	19.55 (5.63)	1.07	0.2 (−0.18 to 0.57)	0.07
Intervention group, mean (SD)					
PHQ-9 score	8.10 (4.83)	5.53 (3.56)	2.57	0.6 (0.08 to 1.12)	0.006
GAD-7 score	6.47 (3.57)	5 (3.24)	1.47	0.43 (−0.08 to 0.94)	0.04
PSS score	18 (4.42)	15.47 (6.29)	2.53	0.47 (−0.05 to 0.98)	0.01

a PHQ-9: Patient Health Questionnaire-9.

b GAD-7: Generalized Anxiety Disorder Assessment-7.

c PSS: Perceived Stress Scale.

To account for the group effect and control for possible residual confounding, 3 ANCOVA models were performed. In each model, the outcome was the depression, anxiety, or stress score obtained after the intervention, and the factors were the study group and the baseline measure corresponding to each outcome. Table 4 shows that the differences between the control and intervention groups for depressive and anxiety symptoms remain significant and similar to those obtained in Table 2. Although the stress difference no longer reached statistical significance. Considering the correlation between depression, anxiety, and stress scores, a multivariate ANCOVA model was applied. Table 4 shows the differences predicted by the model and the differences between the groups in all scores are smaller compared to the bivariate analysis in Table 1. However, they still show that participants in the intervention group had lower values for depressive symptoms, anxiety, and stress. In addition, all these differences have a significant *P* value (*P*<.05), and the effect measures are still classified as having a large effect in favor of the intervention.

**Table 4.** Analysis of variance for comparison of intervention and control groups.

Statistical models and outcomes		*F* test (*df*)	Difference between control and intervention group	ε²_p_^a^	*P* value
ANCOVA^b^					
PHQ-9^c^		9.78 (1, 82)	2.67	0.31	.002
GAD-7^d^		8.28 (1, 82)	2.09	0.32	.005
PSS^e^		3.73 (1, 82)	4.08	0.46	.06
Multivariate ANCOVA					
PHQ-9^c^		7.23 (4, 12)	2.214	N/A^f^	.02
GAD-7^d^		7.23 (4, 12)	1.527	N/A	.04
PSS^e^		7.23 (4, 12)	2.216	N/A	.045

a ε²_p_: squared epsilon effect measure.

b ANCOVA: analysis of covariance.

c PHQ-9: Patient Health Questionnaire-9.

d GAD-7: Generalized Anxiety Disorder-7.

e PSS: Perceived Stress Scale.

f N/A: not applicable.

Assessment of satisfaction, usability, and subjective commitment of the university community in relation to the use of the digital mental health service, several results were shown (Table 5). We observed low satisfaction with the digital service (<24 points on the CSQ-8 scale) in the sample. In addition, more than half of the participants (60%, 18/30) reported a low usability of the platform (<64 points on the CSUQ questionnaire). On the other hand, most participants (96.7%, 29/30) showed a high level of subjective commitment to this tool (≥18 points on the TWEETS questionnaire).

**Table 5.** Level of satisfaction, usability, and subjective commitment to the use of the mental health platform.

Implementation outcomes	Low, n (%)		High, n (%)	
Satisfaction level (CSQ-8)^a^^,^^b^	<24		≥24	
Participant, n (%)				
Administrative staff	1 (100)		0 (0)	
Teacher	6 (100)		0 (0)	
Student	23 (100)		0 (0)	
Total	30 (100)		0 (0)	
Usability level (CSUQ)^a^^,^^c^	<64		≥64	
Participant, n (%)				
Administrative staff	1 (100)		0 (0)	
Teacher	5 (83.3)		1 (16.7)	
Student	12 (52.2)		11 (47.8)	
Total	18 (60)		12 (40)	
Level of commitment (TWEETS)^a^^,^^d^	<18		≥18	
Participant, n (%)				
Administrative staff	0 (0)		1 (100)	
Teacher	1 (16.7)		5 (83.3)	
Student	0 (0)		23 (100)	
Total	1 (3.3)		29 (96.7)	

a Cut-off points determined according to the mean score in each of the questionnaires.

b CSQ-8: Client Satisfaction Questionnaire-8.

c CSUQ: Computer System Usability Questionnaire.

d TWEET: Twente Engagement with Ehealth Technologies Scale.

## Discussion

### Principal Findings

This pilot study assessed the effectiveness of a digital mental health self-care intervention grounded in a contextual-behavioral framework that included acceptance and commitment techniques, experiential avoidance, and mindfulness among students, teachers, and administrative staff at a public university in Peru. Our results suggested that participants in the intervention group experienced a significant reduction in symptoms of depression, anxiety, and stress compared to the control group. Although most participants exhibited high commitment to the platform, satisfaction and usability ratings were low.

Several studies have reported heterogeneous success rates of virtual platforms for mental health and varying user perceptions [34], which in some cases contrasts with our findings. These differences may stem from the therapeutic techniques and approaches used, the duration and format of the intervention, the digital skills of the users, the cultural appropriateness of the intervention, and support personnel.

First, our intervention was grounded in a contextual-behavioral framework, using ACT and mindfulness techniques. Prior studies have demonstrated the effectiveness of these techniques in digital environments. One study assessed an online intervention combining mindfulness and CBT techniques, which showed reductions in depression and anxiety among university students but no significant impact on perceived stress [35]. Another online ACT-based intervention among university students found significant improvements in depression, anxiety, and stress [36]. Together, this evidence suggests that, although mindfulness and CBT techniques effectively address depression and anxiety, complementing them with ACT may be necessary for effective stress management in the university population.

Intervention duration and intensity also influence outcomes. For example, an evaluation of a 6-module digital mental health program found no significant effects on depression and anxiety symptoms [37]. The lack of effectiveness was attributed to low participation and adherence rates, as well as potential issues with intervention delivery. Conversely, another study has indicated that longer interventions (>8 weeks) tend to be more effective in reducing depression and anxiety symptoms [38]. This suggests a need to balance the length of DMHI, tailoring them to specific contexts to achieve effectiveness without hindering user participation. In our study, the reduced module count and diverse content likely supported engagement and contributed to observed mental health improvements, despite overall low completion rates.

Various DMHI formats exist for university students; among these, internet-based interventions are the most commonly used, and our study confirmed their effectiveness in reducing depression and anxiety. However, interactivity and usability are crucial for ensuring this effectiveness [39]. Although our virtual platform featured a responsive design for mobile access, reported satisfaction and usability levels were low, suggesting it failed to provide an optimal user experience to foster participation and maximize benefits. Limited digital skills likely further hindered engagement, as some struggled with navigation, reducing the effective “dose.” In the Peruvian university context, teachers typically possess basic digital skills, and students generally have intermediate skills [40]. These findings partially explain the challenges faced by participants in using the DMHSS, contributing to their low levels of interest, participation, and overall satisfaction.

Several mechanisms may explain why participants maintained high engagement yet rated usability and satisfaction poorly. DMHI may unintentionally create cognitive overload when many modules are displayed simultaneously, a phenomenon linked to “digital fatigue” and reduced app acceptability among university students [41]. Reviews further show that static, nonpersonalized content lowers perceived relevance, whereas adaptive pathways that tailor material to baseline symptom severity improve adherence and satisfaction [42]. Finally, the absence of real-time feedback loops, such as progress indicators or light gamification, diminishes users’ sense of accomplishment; interventions that embed these features consistently achieve high usability scores [43].

In light of this evidence, future platform iterations should streamline navigation via progressive disclosure, incorporate rule-based personalization, and add succinct progress feedback or gamified cues. These refinements, grounded in contemporary usability research, warrant formal A/B testing to quantify their impact on satisfaction, usability, and mental health outcomes, particularly in low-resource university settings. In addition, exploring the incorporation of asynchronous peer-support chats could further assist users with low digital literacy without compromising the autonomy of the intervention.

It has also been emphasized that cultural and contextual aspects should be included in designing DMHI for university students to improve acceptance and effectiveness [44]. In our study, formative studies that assessed the expectations and preferences of students, teachers, and administrative staff regarding DMHI facilitated the intervention design. Including contextually relevant examples and content adapted to the reality of participants likely contributed to the high level of commitment observed despite technological usability or satisfaction challenges.

Moreover, some studies show that support personnel can enhance DMHI effectiveness compared to purely self-administered formats [45], though others find no significant effect [46]. Although our intervention was self-administered, administrative staff provided reminders and technical support, underscoring the value of human support. This highlights the importance of support even in self-administered interventions, particularly among users with procrastination tendencies, which have been shown to reduce the adherence to digital interventions [47], and in contexts of low digital literacy and persistent mental health stigma, such as observed in the Andean region of Peru [7].

Considering the broader applicability of the DMHSS, future integration into existing university mental health services could enhance its impact. Positioning this platform as a first-line self-guided option within a stepped-care model or as an adjunct to traditional counseling services may address unmet mental health needs on campuses. Effective implementation, however, will require adequate IT infrastructure, minimal staff training, clear data-privacy protocols, and consideration of potential barriers such as competing academic demands and limited technical support. Evaluating these factors through future pilot programs is critical to ensuring scalable adoption and sustainability.

### Limitations and Strengths

Several limitations warrant careful consideration. First, attrition was high: only about 20% of intervention participants completed all modules, which narrowed our analytic sample and may limit the representativeness of the findings (external validity). However, the subset who completed the intervention remained balanced at baseline (Table 2) and yielded sufficient power to detect statistically and clinically meaningful between-group differences, suggesting that internal validity was preserved despite the high dropout. Second, completion rates differed between arms (15% intervention vs 25% control), raising the possibility of differential bias. Nevertheless, baseline characteristics (including age, sex, and initial symptom severity) were equivalent across groups (Table 2), suggesting that this imbalance is unlikely to compromise internal validity. Third, satisfaction and usability scores were low, particularly among administrative and teaching staff, underscoring the need for interface refinements. Fourth, all outcomes were self-reported, introducing potential response and social-desirability bias, though validated instruments and blinded allocation mitigate this risk. Fifth, outcomes were measured only immediately postintervention; thus, durability is unknown and longer follow-up is needed. Sixth, we did not conduct qualitative interviews or focus groups; specific cultural and technological barriers were not explored in depth. Future research will incorporate semistructured interviews and user feedback sessions to address these gaps. Seventh, although we report Cohen *d* and ε²_p_ to convey effect magnitude, we did not prespecify or validate minimal clinically important difference thresholds for these instruments, which would aid interpretation of clinical relevance; future work should incorporate such benchmarks. Finally, the study occurred at a single public university in the Andean region, limiting generalizability, although the setting represents typical low-resource universities in the region.

Balanced against these constraints are several noteworthy strengths. The RCT design minimizes selection bias and supports robust causal inference. The intervention is multicomponent and theory-driven, combining ACT, mindfulness, and experiential-avoidance principles that have strong empirical support. In addition, this study extends the scant literature of DMHI in Latin American low-resource contexts [48], and is to our knowledge, one of the first to include teaching and administrative staff alongside students in the effectiveness of digital interventions in mental health in teaching staff, enlarging the scope of evidence on campus mental health of this population [49].

### Recommendations for Future Research

Future studies should evaluate long-term intervention effects with larger samples and replicate research across diverse universities and cultural contexts, including multicountry RCT [50] to assess feasibility, sustainability, and scalability. They should also examine factors influencing participation and engagement, the impact of personalized digital interventions tailored to individual needs, and ways to optimize usability via user-centered, minimalist design, iterative interface evaluation, and enhanced prototypes. It is also recommended to investigate the determinants of low satisfaction, including discrepancies between user expectations and offered functionalities, and the potential role for complementary human support. Future research should use rigorous methodologies such as A/B testing to quantify the effects of these design enhancements on usability, satisfaction, and mental health outcomes in low-resource university settings. Finally, because limited digital literacy may have attenuated our observed effects, future iterations should include targeted digital skills training or onboarding modules to ensure all users can effectively navigate and benefit from the platform.

### Conclusions

In conclusion, this pilot study provides preliminary evidence that a multicomponent intervention of a digital self-care intervention based on acceptance and commitment techniques, experiential avoidance, and mindfulness effectively reduces depression, anxiety, and stress among university populations. This highlights the potential of digital interventions to address mental health problems in populations where access to traditional health services is limited. However, intervention format, content, duration, and level of digital skills of users must be carefully considered to ensure adequate usability and satisfaction, promoting future scalability and sustainability.

## Supplementary material

10.2196/71465 Multimedia Appendix 1 Detailed content and resource breakdown of the 6 DMHSS (digital mental health self-care service) modules.

10.2196/71465 Checklist 1 CONSORT-EHEALTH checklist (V 1.6.1).

## Abbreviations

ACT: acceptance and commitment therapy
ANCOVA: analysis of covariance
CBT: cognitive-behavioral psychotherapy
CONSORT-EHEALTH: Consolidated Standards of Reporting Trials of Electronic and Mobile Health Applications and Online Telehealth
CSQ-8: Client Satisfaction Questionnaire-8
CSUQ: Computer System Usability Questionnaire
DMHI: digital mental health intervention
DMHSS: digital mental health self-care service
GAD-7: Generalized Anxiety Disorder-7
PHQ-9: Patient Health Questionnaire-9
PSS-10: Perceived Stress Scale-10
RCT: randomized controlled trial
TWEETS: Twente Engagement with Ehealth Technologies Scale
