# Correction: Effectiveness, Usability, and Satisfaction of a Self-Administered Digital Intervention for Reducing Depression, Anxiety, and Stress in a University Community in the Andean Region of Peru: Randomized Controlled Trial

**DOI:** 10.2196/87717

**Published:** 2025-12-22

**Authors:** Rosario Yslado-Méndez, Stefan Escobar-Agreda, David Villarreal-Zegarra, Wilfredo Manuel Trejo Flores, Junior Duberli Sánchez-Broncano, Ana Lucia Vilela-Estrada, Jovanna Hasel Olivares Córdova, C Mahony Reategui-Rivera, Claudia Alvarez-Yslado, Leonardo Rojas-Mezarina

**Affiliations:** 1 Facultad de Ciencias Médicas, Universidad Nacional Santiago Antúnez de Mayolo, Av. Centenario 200, Huaraz, 02002, Peru, +51 042640020; 2 Unidad de Telesalud, Facultad de Medicina, Universidad Nacional Mayor de San Marcos, Lima, Peru; 3 Digital Health Research Center, Instituto Peruano de Orientación Psicológica, Lima, Peru; 4 Universidad Científica del Sur, Lima, Peru; 5 Facultad de Ciencias, Universidad Nacional Santiago Antúnez de Mayolo, Huaraz, Peru; 6 Escuela de Posgrado, Universidad San Ignacio de Loyola, Lima, Peru; 7 Facultad de Ciencias Sociales, Educación, Comunicación, Universidad Nacional Santiago Antúnez de Mayolo, Huaraz, Peru

In “Effectiveness, Usability, and Satisfaction of a Self-Administered Digital Intervention for Reducing Depression, Anxiety, and Stress in a University Community in the Andean Region of Peru: Randomized Controlled Trial” [1], the authors made several adjustments.

The corresponding author’s phone number was changed to the following:

51 042640020

The affiliation for authors RY-M, JDS-B, and JHOC has been changed from the following:

Facultad de Ciencias Sociales, Educación, Comunicación, Universidad Nacional Santiago Antúnez de Mayolo, Huaraz, Peru

Their affiliation now reads as:

Facultad de Ciencias Médicas, Universidad Nacional Santiago Antúnez de Mayolo, Huaraz, Peru

This has resulted in the change of affiliation 1. As author CA-Y is still affiliated with the original affiliation, it remains in the affiliation list as affiliation 7.

The affiliation for WMTF has been changed from:

Facultad de Ciencias Sociales, Educación, Comunicación, Universidad Nacional Santiago Antúnez de Mayolo, Huaraz, Peru

To read:

Facultad de Ciencias, Universidad Nacional Santiago Antúnez de Mayolo, Huaraz, Peru

Additionally, affiliation 4 has been added to the affiliations for ALVE. All other affiliations have been renumbered according to these amendments.

The correction will appear in the online version of the paper on the JMIR Publications website, together with the publication of this correction notice. Because this was made after submission to PubMed, PubMed Central, and other full-text repositories, the corrected article has also been resubmitted to those repositories.
