# Supplementary material for: Effectiveness, Usability, and Satisfaction of a Self-Administered Digital Intervention for Reducing Depression, Anxiety, and Stress in a University Community in the Andean Region of Peru: Randomized Controlled Trial
Source: JMIR Form Res. 2025 Oct 15;9:e71465. doi: 10.2196/71465 (PMC12530451; doi:10.2196/71465)
Supplement: Multimedia Appendix 1 [file formative-v9-e71465-s001.docx]

**Table S1.**

The following table summarizes each module’s objectives, number and type of videos, daily messages, PDF workbooks, and audio files.

| **Module** | **Objectives** | **Video Content** | **Message Content** | **PDF Workbook** | **Audio Content** |
| --- | --- | --- | --- | --- | --- |
| **1. Acceptance & Commitment (ACT)** | • Cultivate acceptance of difficult emotions and thoughts associated with depression.  • Learn to recognize and accept anxiety instead of fighting it.  • Identify stressors and ineffective coping responses. | • 1 FLV video: Introduction to ACT and its application to depression, anxiety, and stress.  • 1 FLV video: Examples of cultivating acceptance of difficult emotions/thoughts.  • 1 FLV video: Strategies to recognize and accept anxiety vs. struggle.  • 1 FLV video: Identifying stressors and exploring ineffective coping. | • 10 daily reminders to practice acceptance of difficult emotions/thoughts.  • 10 reflective prompts on when to accept anxiety rather than control it.  • 10 self-reflection questions to identify stressors and coping responses. | 3–8 pages, color, PDF A4 step-by-step guide to practicing emotional acceptance | 4 MP4 audio tracks mirroring video content |
| **2. Mindfulness** | • Introduce mindfulness practice and its relation to depression, anxiety, and stress.  • Practice nonjudgmental observation of depressive thoughts and emotions.  • Explore breathing and relaxation techniques for physiological regulation | • 1 FLV video: Mindfulness introduction and relevance.  • 1 FLV video: Mindful observation exercises for depressive thoughts/emotions.  • 1 FLV video: Breathing and relaxation techniques (anxiety regulation).  • 1 FLV video: Meditation practices for calm and balance. | • 10 reminders to practice mindfulness and observe thoughts/emotions nonjudgmentally.  • 10 reflections on cultivating calm/balance in stressful situations. | 3–8 pages, color, PDF A4 mindfulness exercises worksheet | 4 MP4 audio tracks mirroring video content |
| **3. Experiential Avoidance** | • Explore common avoidance strategies and their impact on depression.  • Provide examples of confronting feared situations adaptively.  • Develop emotional self-regulation skills to tolerate stress | • 1 FLV video: Overview of avoidance strategies and depression.  • 1 FLV video: Examples of adaptive confrontation of feared situations.  • 1 FLV video: Emotional regulation techniques for stress tolerance. | • 10 reflections on avoidance strategies and their link to depression.  • 10 guidelines for gradual fear confrontation and avoidance reduction.  • 10 tips for emotional self-regulation and stress management. | 3–8 pages, color, PDF A4 worksheets to identify stressors and coping patterns | 3 MP4 audio tracks mirroring video content |
| **4. Values Clarification (ACT)** | • Identify and clarify personal values as a guide for change and recovery | • 1 FLV video: Values identification and clarification.  • 1 FLV video: Value-based adaptation strategies (e.g., healthy boundaries, social support, self-care). | • 10 reflections on the importance of personal values for motivation.  • 10 prompts on using values in stressful contexts. | • 3–8 pages, color, PDF A4 exercises to set values and life plans | • 4 MP4 audio tracks mirroring video content |
| **5. Self-Compassion & Self-Care** | • Cultivate self-compassion and personal care for emotional well-being.  • Foster non-reactivity toward anxious thoughts and sensations.  • Challenge rumination patterns to enhance mental clarity. | • 1 FLV video: Self-compassion and self-care practices.  • 1 FLV video: Mindfulness exercises for accepting anxious thoughts/sensations.  • 1 FLV video: Explaining the link between stress thoughts, emotions, and rumination. | • 10 reflections on self-compassion’s role in recovery.  • 10 tips for acceptance and non-reactivity toward anxiety.  • 10 strategies to challenge rumination and worry. | 3–8 pages, color, PDF A4 practical exercises for self-compassion and self-care | 3 MP4 audio tracks mirroring video content |
| **6. Behavioral Activation** | • Promote gradual exposure to pleasurable and meaningful activities to counter anhedonia and restore positive emotion capacity | • 1 FLV video: Importance of gradual pleasurable activity exposure.  • 1 FLV video: Examples of meaningful activities for gradual practice.  • 1 FLV video: Demonstrations of effective coping techniques. | • 10 suggestions for pleasurable, meaningful activities to practice gradually.  • 10 tips for using effective coping strategies in stressful situations. | 3–8 pages, color, PDF A4 planning and recording worksheets for exposure and coping | 3 MP4 audio tracks mirroring video content |
